# Benchmark data set for breast cancer associated genes

**DOI:** 10.1016/j.dib.2022.108583

**Published:** 2022-09-13

**Authors:** Sushrutha Raj, Athira P Anil, Anshita Shukla, Kadambala Anoosha, Alok Srivastava

**Affiliations:** a Amity Institute of Integrative Sciences and Health, Amity University Haryana, Amity Education Valley, Gurgaon, 122413, India; b Institute of Bioinformatics and Computational Biology, Visakhapatnam, Andhra Pradesh, 530017, India; c Sri Innovation and Research Foundation, Ghaziabad, 201009, India; d L V Prasad Eye Institute, Hyderabad, Telangana 500034, India

**Keywords:** Breast cancer genes, Double cross validation, Meta-analysis, System modeling, Text mining, Machine learning, Text classifier, Breast cancer association

## Abstract

Breast cancer is one of the leading causes of death in women worldwide. The main reason could be inheritance, change in environmental conditions or the mutation in certain genes that cause cancer. These genes are not negligible, on the contrary, a wide range of genes have their involvement in the development and progression of different stages of breast cancer. In this article, we are going to explore the association of breast cancer genes and classify them into different association classes viz. positive, negative and neutral. Among all the available biomedical literature resources for a disease, HuGE Navigator is a major resource comprising continually updated human genome epidemiology data controlled by the Centers for Disease Control and Prevention. However the literature finder module of HuGE Navigator only yields PubMed IDs for a specific disease, which are explored further to retrieve abstract data from PubMed. These abstracts are filtered out to include those reference sentences which have at least one gene and disease term. This reference sentence data has been taken as a reference to apply double-fold cross-validation to compile the most comprehensive list and then classify them into different association classes viz, positive, negative or neutral along with the reference sentences confirming the association of the disease with the gene. The positively associated data generated here can be used for breast cancer modelling or meta-analysis of breast cancer. The data generated in the present work can be used as standard reference data for the training of text mining-based biological literature classifiers to predict the class of published literature not only in breast cancer but in other diseases as well.

**Specifications Table**

**Table:** 

Subject	Health Informatics
Specific subject area	Breast cancer genes, Breast cancer gene classification, Text classification
Type of data	Tables
How data were acquired	Raw data was downloaded from HuGE Literature Finder via https://phgkb.cdc.gov/PHGKB/startPagePubLit.action
Data format	Raw, Processed, and Analyzed
Parameters for data collection	Raw data collected contains disease name, disease class, gene, PubMed id, association class, title of article, year of publication and reference sentence supports the association.
Description of data collection	Raw data collected from an archives of published genetic association studies that contains information on molecular and clinical parameters.
Data source location	https://phgkb.cdc.gov/PHGKB/startPagePubLit.action
Data accessibility	Data are available on Mendeley data source via https://data.mendeley.com/datasets/xdkvk75ns7/2

## Value of the Data

• The data validated in this article would be useful as a reference dataset to train the text based biological literature to build a machine learning classifier. The classifier will be further used to predict the class of published literature into positive, negative and neutral classes not only in the case of breast cancer, but also in other diseases as well. This classified dataset can further be explored to identify breast cancer genes classified into different association classes. These association classes are supported by weight criteria based on the empirical probability for each class.
• This data will be helpful for the investigators working in the domain of breast cancer for the system-level modelling of the disease as well as to develop a deeper understanding of the breast cancer biomarkers. This dataset will have a wider application for the researcher working in the area of natural language-based classifiers to classify the text irrespective of disease.
• There are 419 ambiguous genes in the breast cancer association class, whose association is not properly defined. These genes can be used to plan further experiments to confirm their association with breast cancer. As few genes are annotated on the multiple association classes, these genes can be further used to find strong evidence for their association classes with the disease.
• Positive associated breast cancer genes can be further explored to study their roles in specific subtypes of breast cancer, their metastasis study based on common genes associated with other disease, as well as for system level modeling, meta-analysis of disease to study the differential evolution of the genes impact the disease.

## 1 Data Description

The raw data referenced for this study is described in detail in raw data description. This data is further validated manually to avoid any possibility of superfluous data and then further be used for classification of its association with the genes into multiple associative classes such as positive, negative and neutral classes. The method used for processing of this raw data is double fold cross validation. This method discarded false predictions and generated a processed file that is used to calculate weight of individual gene association classes. We have discussed the above-mentioned data in detail in the preceding sections.

### 1.1 Raw Data Description

There are numerous sources of biomedical data available to extract genes associated with specific diseases. Among several such resources, Human Genome Epidemiology (HuGE) Navigator [1] gives access to an up-to-date knowledge database that comprises information on population prevalence of genetic variants, gene-disease associations, gene-gene and gene- environment interactions, and evaluation of genetic tests. Since the HuGE literature finder returns only the PubMed IDS related to a specific disease, we further used EDirect [2] database to extract abstract text data of these PubMed IDs. Edirect is a tool developed by NCBI used to retrieve data from Pubmed. The summary of the extracted abstracts data is included in Table 1.

**Table 1:** Summary of raw breast cancer data filtered using HuGE Literature Finder. Unique class represents one of the association classes that belongs to the type of association between breast cancer and genes.

Entities	Number
Duration	1987-2021
Unique number of genes	1761
Unique classes reported	3
Unique PubMed ID	4627
No of entries	12565

Edirect is further used to extract and map the required fields to this abstracts text data. The details of each field extracted are included in Table 2. This raw data of breast cancer used for study has been included in Supplementary 1.

**Table 2:** Description of data extracted from EDirect for Breast Cancer query.

Field Name	Description
DISEASE	Disease Name
DIS_CLASS	Disease Class
GENE	Gene Symbol
PUBMED ID	PubMed ID of the article
LACKASSO	Type of Association
TITLE	Title of the article
YEAR	Year of Publication
CONCLUSION	Reference sentence

### 1.2 Preprocessing of Raw Data

Supplementary 1 containing 12565 records is processed to avoid anomalies, duplicate entries and redundancy from the data. Summary of entries removed are mentioned in Table 3.

**Table 3:** Summary of redundant entries removed from raw data to clean the data.

Category	Field Processed	Count
Duplicated entries	All	41
Genes not available	Gene	32
Gene Name not applicable	Gene	1033
“variant(s)” as Gene Symbol	Gene	2
“NA” as Gene Symbol	Gene	2
“intergenic” and its variants as Gene Symbol	Gene	1
Non protein coding gene	Gene	1
Records needs to validate	-	11453

**Total**		**12565**

After processing Supplementary 1, there were 12565 records left which were processed further. This data has been further double fold manually validated to rectify the false information from the raw data. Summary of data classified into different association classes is described in Table 4. However, genes included in this data mentioned in Table 4 are overlapped with multiple association classes.

**Table 4:** Summary of filtered breast cancer data classification into various association classes.

LACKASSO	No of Entries	No of genes
Y	296	136
N	331	190
B	3531	827
Not reported	8407	1182

**Total**	12565	2335
**Unique genes**		1761

This data has been further double-fold manually validated to rectify the false information from the raw data.

## 2 Experimental Design, Materials and Methods

### 2.1 Data Extraction

Our aim in the present work is to create a benchmark disease gene association dataset for breast cancer by thoroughly validating the existing disease-gene association data. We have used HuGE Navigator to extract the PubMed Ids related to breast cancer. We found 7073 PubMed Id between 1987 to Dec 2021 related to breast cancer. Further, in order to extract the abstract data corresponding to these PubMed IDs, we used the NCBI's EDirect application. The details of the abstracts data extracted from EDirect and the query used are mentioned in Table 5 below.

**Table 5:** Summary of breast cancer specific abstracts data downloaded.

**Disease**	Breast Cancer
**Source**	edirect
**Date of download**	17/01/22
**Query used**	cat table_of_pubmed_ids |epost -db pubmed |efetch -format xml | xtract -pattern PubmedArticle -sep " " -tab "_%%%%_" -element PUBMED ID AbstractText
**Query Count (Abstracts)**	7073
**Sentences containing atleast one gene and disease name**	12565

In the next step, this abstract data was manually filtered to include those reference sentences which have at least one gene and disease term together. We could extract 12565 reference sentences with least one disease and gene term together in 7073 abstracts.

### 2.2 Data Validation

12565 data entries have been processed to create a benchmark dataset for breast cancer.

A Type 1 error or false positive error also known as false positive indicates a given condition exists when actually it does not and gives an incorrect positive decision. Whereas a Type 2 error or false negative error, which is also known as false negative wrongly indicates a condition that does not hold true. In the present context of association between the disease and gene in a scientific literature, false positives are those in which positive association is indicated in the raw data (LACASSO field) but in reality, no association between the mentioned GENE and DISEASE exists. Whereas false negatives are those where negative association is indicated in the raw data when actual association exists.

In order to remove the false positives and false negatives from the raw data processed in Supplementary 1, we have applied double fold manual validation for each record. We have validated the reference sentence mentioned in CONCLUSION field with respect to the association class mentioned in LACASSO field with the mentioned gene in the GENE field. We have kept the true positive and true negative records, while those having ambiguity are further cross checked and updated from the same reference. In case of references that do not have a clear association mentioned between the disease and gene, we classified them into several other sub categories mentioned in ASSOCIATION.CLASS as described in Table 6.

**Table 6:** Distribution of sub categories of ASSOCIATION_CLASS of breast cancer processed data.

S No	Sub category	Description	Count
1	Y	Gene is positively associated with Breast cancer disease	2513
2	N	Gene is not associated with breast cancer disease	1019
3	A	Gene is ambiguous in nature. Word “may” or “may not” is used to define the association of genes with Breast cancer disease	1635
4	MC	Mis Classified. Multiple entries for which PubMed ID, gene symbol, reference sentences are the same, but classification are different.	8
5	ANF	Abstract Not Found. For the given PubMed ID abstract is not found in the reference article.	45
6	D	Duplicate entries. For multiple reference genes, reference sentences, PubMed ID and association class are the same.	0
7	NR	Not Related. Abstract is not related to Alzheimer	87
8	PNF	PubMed ID Not Found	1
9	X	Abstract does not contain any information about the association between mentioned gene in GENE field and Breast cancer	6145

		**Total**	**11453**

### 2.3 Mapping of Gene names to Approved Gene symbols

The gene description mentioned in the REF_GENE field contains name, synonym, symbol or alias name. These terms have been mapped to the approved gene symbol given in HUGO Gene Nomenclature Committee (HGNC) [3]. The approved gene symbol mapped with the other synonymous or alias terms are updated in the GENE_NEW field of the processed data.

### 2.4 Computation of Association weights

The processed data contains multiple entries for the same gene in the GENE_NEW field; this group of genes is defined as a gene block. A gene block can have multiple association classes. In such cases the empirical probability for each association in a gene block is calculated. This probability defines the score of the association class of a gene with breast cancer. The processed data file is updated with six new fields defined in Table 7.

**Table 7:** Description of fields added to the raw data.

S No	Field Name	Description
1	DB.ID	Unique entry ID
2	REF.SENTENCE	Validated reference sentence mentioned for disease gene association
3	ASSOCIATION.CLASS	Association class of validated reference sentence
4	REF.GENE	Gene symbol mentioned in the reference sentence. It could be a gene symbol, gene name, aliases or synonymous terms for the referred gene.
5	GENE.NEW	Approved gene symbol of REF_GENE field, used to calculate WEIGHT
6	WEIGHT	Disease gene association probability

### 2.5 Allotment of Association class

Using maximum weight obtained for each association class for a given gene group, a particular association class was then assigned to the gene. This information of computed weights has been provided in Supplementary 2. The data we have prepared contains individually validated information on genes in connection with breast cancer with supporting reference. This data can be used as a benchmark data set for the association of Breast Cancer with the genes. Further, one can choose all the genes annotated to positive class using the weight criteria. This data can also be used for training a machine learning algorithm for classification of disease gene associations in the scientific literature. Further this data can be combined with other similar data on other diseases [4], that will increase the size of the data to train the machine learning model to improve the classification performance. The genes mentioned as positive association can be further explored to do the system level modeling of breast cancer.

## Ethics Statements

This study involves neither humans nor animals. The authors declare that this submission follows the policies as outlined in the Guide for Authors and in the Ethical Statement.

## Supplementary Materials

Supplementary material associated with this article can be found, in the online version, at https://data.mendeley.com/datasets/xdkvk75ns7/2

## CRediT authorship contribution statement

**Sushrutha Raj:** Methodology, Validation, Writing – original draft, Supervision. **Athira P Anil:** Validation, Writing – original draft. **Anshita Shukla:** . **Kadambala Anoosha:** Validation, Writing – original draft. **Alok Srivastava:** Conceptualization, Methodology, Writing – original draft, Writing – review & editing, Supervision, Project administration, Funding acquisition.

## Declaration of Competing Interest

The authors declare that they have no known competing financial interests or personal relationships that could have appeared to influence the work reported in this paper.

## Data Availability

Benchmark gene reference data for Breast Cancer (Reference data) (Mendeley Data).
